# Leveraging Digital Twins for Stratification of Patients with Breast Cancer and Treatment Optimization in Geriatric Oncology: Multivariate Clustering Analysis

**DOI:** 10.2196/64000

**Published:** 2025-05-23

**Authors:** Pierre Heudel, Mashal Ahmed, Felix Renard, Arnaud Attye

**Affiliations:** 1 Department of Medical Oncology, Centre Leon Bérard, 28 rue Laennec, Lyon, 69008, France, 33 478782952; 2 GEODAISICS, Grenoble, France

**Keywords:** digital twins, artificial intelligence, breast cancer, older adult patients with cancer, treatment, geriatric oncology, geriatric, oncology, cancer, clustering analysis, therapeutic, older adult, elder, old, patients with cancer, decision-making tools, decision-making, manifold learning model, chemotherapy, comorbidities, comorbidity, health care

## Abstract

**Background:**

Defining optimal adjuvant therapeutic strategies for older adult patients with breast cancer remains a challenge, given that this population is often overlooked and underserved in clinical research and decision-making tools.

**Objectives:**

This study aimed to develop a prognostic and treatment guidance tool tailored to older adult patients using artificial intelligence (AI) and a combination of clinical and biological features.

**Methods:**

A retrospective analysis was conducted on data from women aged 70+ years with HER2-negative early-stage breast cancer treated at the French Léon Bérard Cancer Center between 1997 and 2016. Manifold learning and machine learning algorithms were applied to uncover complex data relationships and develop predictive models. Predictors included age, BMI, comorbidities, hemoglobin levels, lymphocyte counts, hormone receptor status, Scarff-Bloom-Richardson grade, tumor size, and lymph node involvement. The dimension reduction technique PaCMAP was used to map patient profiles into a 3D space, allowing comparison with similar cases to estimate prognoses and potential treatment benefits.

**Results:**

Out of 1229 initial patients, 793 were included after data refinement. The selected predictors demonstrated high predictive efficacy for 5-year mortality, with mean area under the curve scores of 0.81 for Random Forest Classification and 0.76 for Support Vector Classifier. The tool categorized patients into prognostic clusters and enabled the estimation of treatment outcomes, such as chemotherapy benefits. Unlike traditional models that focus on isolated factors, this AI-based approach integrates multiple clinical and biological features to generate a comprehensive biomedical profile.

**Conclusions:**

This study introduces a novel AI-driven prognostic tool for older adult patients with breast cancer, enhancing treatment guidance by leveraging advanced machine learning techniques. The model provides a more nuanced understanding of disease dynamics and therapeutic strategies, emphasizing the importance of personalized oncology care.

## Introduction

Breast cancer is more commonly diagnosed in older populations, particularly among women aged 65 years and older in wealthier countries. In the United States, the average age of breast cancer diagnosis is 62 years, and in 2020, women aged 70 years and older accounted for 30% of all new cases of the disease [1,2]. In the European Union, women older than 65 years made up about 44% of all breast cancer cases [3]. However, treatment approaches for early-stage breast cancer in these older age groups are often inadequate and unclear, largely due to a lack of solid evidence and the unreliability of web-based tools for making decisions about additional therapies, leading to less than ideal treatment outcomes [4,5].

The treatment plan for breast cancer is tailored based on the cancer’s characteristics, the patients’ overall health status, and their personal preferences. Standard care for early-stage breast cancer usually involves surgery, and may also include radiation, as well as neoadjuvant or adjuvant systemic therapy, used alone or in various combinations. Crafting postsurgical treatment strategies for older patients with breast cancer is complex due to their typically compromised health and the lack of data from clinical trials, since older adults are seldom participants in such studies and are not well represented in meta-analyses that evaluate the effectiveness of adjuvant chemotherapy in reducing breast cancer mortality and improving survival rates [6,7]. Consequently, artificial intelligence (AI) has been investigated as a potential tool to support decision-making in the context of limited clinical trial evidence.

Early uses of AI in cancer treatment guidance involved knowledge-based systems [8,9]. Recently, a broader spectrum of machine learning methods has been examined to aid both clinicians and patients with breast cancer [10-15]. Nonetheless, most decision support tools are designed for patients aged between 18 and 65 years, reflecting the age group most studied, with limited research focusing on treatment outcomes for older patients with breast cancer [16-19]. The prognostic tool PREDICT [20], although popular, has shown limited effectiveness for older adult patients [21]. Adjutorium [22], which uses extensive datasets from the United Kingdom and the United States, provides more precise prognosis and treatment benefit predictions for breast cancer than PREDICT. Despite this, it primarily includes patients aged between 30 and 65 years, with fewer older patients in its datasets, and omits certain vital tumor information such as progesterone receptor (PR) status [19]. Another established tool, Adjuvant! Online, predicts 10-year overall survival, breast cancer survival, and recurrence rates, commonly used to inform expected outcomes from endocrine therapy and chemotherapy [23]. Its accuracy is questionable for older women with early-stage breast cancer, probably because it was trained on data with a maximum age limit of 69 years [24]. In a review by Engelhardt et al [25], various models could forecast breast cancer outcomes, typically based on genetic risk scores, but only Adjuvant! Online factored in comorbidity status. Yet, none had been thoroughly validated in older adult populations. The more recent PORTRET tool was designed to predict 5-year recurrence, overall mortality, and mortality from other causes in patients older than 65 years with early invasive breast cancer, as well as to estimate the benefits of adjuvant systemic treatment [26]. The tool’s authors observed that their treatment effect estimates were based on data from pooled randomized clinical trials, which might not be entirely applicable to older adults due to the typically selective nature of older participants in these trials.

This study aims to develop models that overcome the shortcomings of past research by using cohorts that accurately reflect the demographic of older patients with breast cancer and by leveraging a detailed dataset that includes administrative, biological, treatment, primary tumor, and survival information. Our latest research uses manifold learning, an advanced tool for nonlinear dimensionality reduction that excels in unraveling complex geometric relationships within high-dimensional data, revealing intricate connections between clinical factors.

We introduce a new prognostic and predictive tool tailored for older adult patients with breast cancer, providing postoperative treatment recommendations. This tool is distinctive in its consideration of the interdependencies among variables within a patient population. It acknowledges the relative importance of prognostic factors in a way that many existing models do not. Our findings are set to be extremely beneficial for oncologists when determining suitable adjuvant treatment approaches for older adult patients with breast cancer, taking into account the nuances of both tumor-related and patient-specific characteristics.

## Methods

### Recruitment

In this retrospective study, we examined pseudonymized data from women aged 70 years and older who received a diagnosis of early-stage breast cancer and underwent surgery with the intent to cure (either lumpectomy or mastectomy, with or without axillary lymph node dissection) at the French Léon Bérard Cancer Center from January 1997 to December 2016. The French Léon Bérard Cancer Center is a 300-bed comprehensive cancer center located in Lyon, France, serving more than 30,000 patients annually, with a multidisciplinary team of 2000 health care professionals and a catchment area covering southeast France.

The inclusion criteria were not limited by the breast cancer’s histological or molecular characteristics, the size of the tumor, or the status of the lymph nodes. However, the study did exclude patients who had noninvasive in situ carcinoma without invasive carcinoma, HER2 (human epidermal growth factor receptor 2) positive breast carcinoma, or who presented with distant metastases at the time of surgery. HER2-positive breast cancer cases were excluded because these patients typically receive trastuzumab-based targeted therapies, which dramatically improved their prognosis following its widespread adoption for nonmetastatic breast cancer around 2005. In contrast, chemotherapy protocols for HER2-negative cases remained consistent during the treatment period of the patients included in this study, ensuring uniformity in therapeutic strategies and outcomes across the cohort. The research concentrated on the 5-year survival rates, selecting only those who had at least 5 years of follow-up and whose vital status information was available.

The database was constructed using ConSore, a data-mining application developed by UNICANCER [27]. The ConSore platform extracts data from the electronic health records of the Léon Bérard Cancer Center, integrating patient demographics, clinical variables, and treatment details. To ensure accuracy, each record was also subject to a manual verification process. Data compiled included demographic details and clinical features of patients at diagnosis, alongside comprehensive biological and disease-specific information, and the treatments administered.

We included the following characteristics for patients diagnosed with early-stage breast cancer: age; Eastern Cooperative Oncology Group performance status; BMI; comorbidities such as diabetes, heart failure, coronary artery disease, chronic obstructive pulmonary disease, and cognitive impairments; history of hospitalizations; and polypharmacy. We also gathered biological indicators at the time of diagnosis, which included hemoglobin levels, lymphocyte counts, and creatinine clearance. We extracted data on disease attributes including histological subtype, hormone receptor status, HER2 status, Scarff-Bloom-Richardson (SBR) grade, tumor count, size of the largest tumor, and the extent of lymph node involvement as per the Tumor,” “Nodes,” “Metastases (TNM) classification [28]. The statuses of estrogen receptors (ERs), PRs, and HER2 were determined from the histopathological analysis of pretreatment biopsies. Hormone receptor negativity was classified when fewer than 10% of cells were stained for ER and PR. HER2 negativity was assigned when immunohistochemistry staining was below 1+. For tumors scoring 2+, further in situ hybridization tests were conducted to assess HER2 amplification [29]. Treatment data collected encompassed the type of surgery performed, lymph node dissection, and adjuvant treatments including radiotherapy, chemotherapy, and endocrine therapy.

### Outcome, Predictors, and Predictive Power

Outcome was overall survival in 5 years. Due to the high percentage of missing values for cause of death, cancer-specific survival was not considered. Nine predictors were selected: age, tumor size (mm), tumor grade (defined as either SBR low: 1‐2; or high: 3), number of affected ganglions, hormone-receptor status (positive if either estrogen or PRs were immunohistochemically present in ≥10% of tumor cells; otherwise, patients were classified as triple negative), serum hemoglobin (g/dL) and lymphocyte count (G/L), BMI, and the presence of comorbidities.

The initial database, built using ConSore, compiled a range of clinical, biological, and disease-specific data, along with information on administered treatments. We aimed for a predictors representing a mixture of features typically tested before patients undergo treatment plans. Thus, we excluded features regarding treatments as (1) we wanted to gauge prediction accuracies based only on the initial testing of the patient, and (2) the efficacy of treatment strategies was also an outcome of interest in the study. We further excluded features with significant number of missing values so as to limit the loss of usable data. Creatinine was excluded due to its high correlation with patient’s age and potential kidney disorders that are not uncommon in the study’s demographic. The feature was found to correlate with negative patient outcome, but this was independent of cancer and introduced a bias. Following these steps, 9 predictors were isolated, a list comprising both continuous and categorical variables, as well as an acceptable mixture of relevant biological and clinical features. Random Forest Classification (RFC) and Support Vector Classifier (SVC) were used to evaluate the predictive power of the selected features. We used 5-fold cross-validation to mitigate overfitting and ensure the validity of our results.

### Model Development and Validation

Patients in the initial cohort with missing values for any of the 9 predictors were cut from the study. The remaining patients comprised the model development cohort. This was divided into reference and model data.

#### Reference Data and Digital Twins

The reference data inclusion criteria were positive outcome for survival in 5 years and remission without relapse by the last follow-up. The purpose of this group was to calibrate the our patented algorithm, generating digital twins for future test subjects. Digital twins refer to synthetic patient data derived from the reference group specifically similar in profile to a new test subject. The model uses these synthetic profiles to recognize complex variations within the test profile. Thus, digital twins are generated and used in the model to provide recommendations on a new patient but do not themselves constitute the result that a physician would need to interpret.

#### Model Data

The model data, distinct from the reference data to prevent data leaking, are the population that is run through the precalibrated model and scored against the reference group. The data are thus transformed from raw patient data to a numerical and standardized representation of their deviation from the reference group (their digital twins). The purpose of these transformed data is to populate the model with a range of patient profiles that will serve for future prognostic analysis.

### PaCMAP, Mean-Shift Clustering, and Manifold Visualization

**Figure 1.**
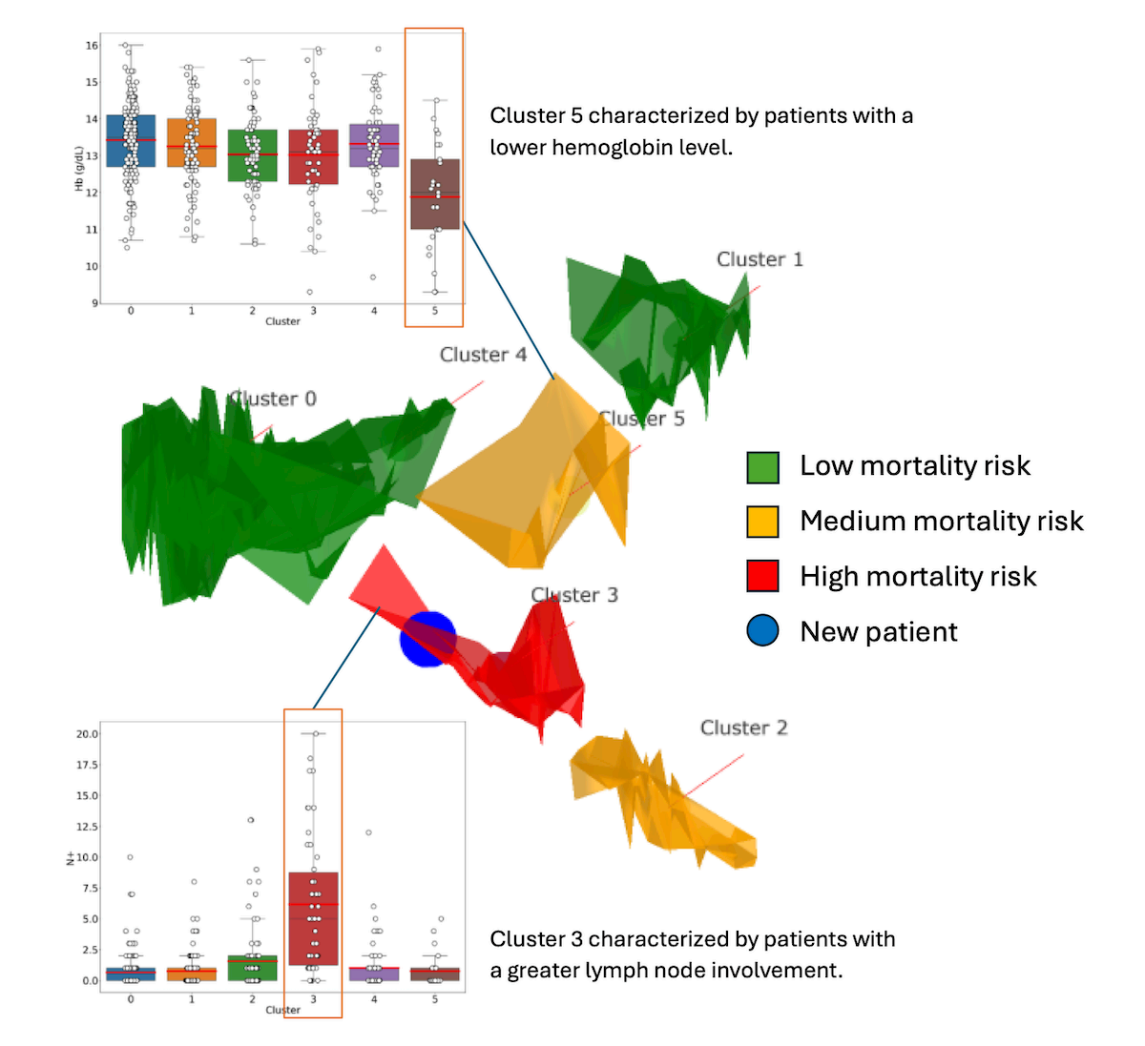
Graphical representation of the 6 clusters of patients in the 3D manifold space. Patients in the reduced 3D space, or manifold, were grouped into clusters by their spatial distribution and profile similarity. Clusters were then colored based on the overall mortality rate of included patients. A newly tested patient is localized on the manifold and represented by a blue sphere.

The transformed model data underwent dimensionality reduction using PaCMAP (Pairwise Controlled Manifold Approximation) [30] to generate 3D data referred to as a manifold, permitting easy visualization. The data were then stratified using mean-shift clustering [31], a nonparametric, density-based clustering algorithm that can be used to identify clusters in a dataset (Figure 1). Each cluster represents a local group of similar patients in the 3D space. Clusters represent typical patient profiles in the overall population. The advantage of clustering is that it captures the variability of subjects of a subgroup for easy analysis. A better understanding of the cluster and its variability allows clinicians to assess whether a new test subject aligns well with the cluster and to identify potential differences. When considering a new patient, estimates of prognosis and expected benefits of adjuvant treatment are ascertained by the examination of cluster-specific treatment outcomes pertinent to the patient’s clinical profile.

### Prediction of Chemotherapy Benefit

To estimate the benefit of chemotherapy, the position of a new patient is identified within the 3D manifold. Using the K-nearest neighbors algorithm, the 15 closest chemotherapy-treated patients and the 15 nearest non–chemotherapy-treated patients are pinpointed. Kaplan-Meier (KM) survival curves were plotted for each of these patient groups, providing a visual estimation of chemotherapy benefit for a clinical profile.

### Validation of Treatment Benefit Predictions With Kullback-Leibler Divergence

To validate that the distributions of the 2 treatment subgroups are comparable, we used the Probability Density Function, which describes the spread of the data points in the 3D space. To measure the difference between these distributions, we applied the symmetrized Kullback-Leibler (KL) divergence, a statistical method that quantifies how much one distribution differs from another. To assess whether the observed difference was meaningful or just due to random chance, we conducted a permutation analysis. This technique works by randomly shuffling the data multiple times to create many new random comparisons; comparing the real result with the random results allows us to determine whether the observed difference between the distributions was statistically significant. If distributions of 2 different treatment groups were found to be similar, they could be compared to provide a prediction of treatment benefit.

### Model Stability Validation

The original model data were split into 2 groups: 70% (327) of every cluster was pooled into the training group, and the remaining 30% (139) was pooled into the test group. A new manifold learning process was applied to the training group, and the test group was then projected onto this newly generated manifold. Patients in the test and model groups from the same cluster of origin were compared to evaluate whether data points would exhibit similar distributions (appear in proximity to each other) in the new manifold space across 10 different manifold initializations.

### Statistical Analysis

#### Kullback-Leibler Divergence and Permutation Test

The symmetrical KL divergence was used to measure the difference between 2 probability distributions. A permutation test was subsequently conducted to assess the significance of the observed KL divergence. This involved calculating the KL divergence for a large number of permutations of the combined datasets and comparing these values with the original KL divergence. The *P* value is calculated as the proportion of permutations where the KL divergence is as extreme as, or more extreme than, the original KL divergence calculated between the actual groups, thus providing a measure of how likely it was to observe a divergence as extreme as the original, under the null hypothesis of no difference between the distributions. Mathematically, this *P* value is the ratio of the number of permuted KL divergences that are equal to the original KL divergence or greater to the total number of permutations. A low *P*-value suggests that the observed difference in distributions is unlikely to have occurred by chance, thus indicating a significant divergence between the 2 groups.

#### Survival Analysis using the KM Estimator and Log-Rank Test

The KM estimator was used to generate survival curves for different treatment subgroups. The log-rank test, a nonparametric test, was applied to compare the survival distributions and a *P* value was calculated to determine the statistical significance of the differences observed between the groups. A low *P* value suggests that the observed survival curves are significantly different. The statistical package used for the analysis is Lifelines 0.30.0 (Lifelines Developers) [32].

### Ethical Considerations

This retrospective study involving human subjects was reviewed and approved by the French data protection authority, the Commission Nationale de l’Informatique et des Libertés, under authorization number 9191415, dated October 10, 2019. According to institutional and national guidelines, no additional approval from a research ethics board was required, as the data used were previously collected for clinical purposes. No new informed consent was required for this study. The analysis was conducted using data for which participants had provided general consent at the time of data collection. All data were pseudonymized prior to analysis to protect patient confidentiality. No identifiable personal information was retained in the research dataset. No compensation was provided to participants.

## Results

### Cohort Characteristics

**Figure 2.**
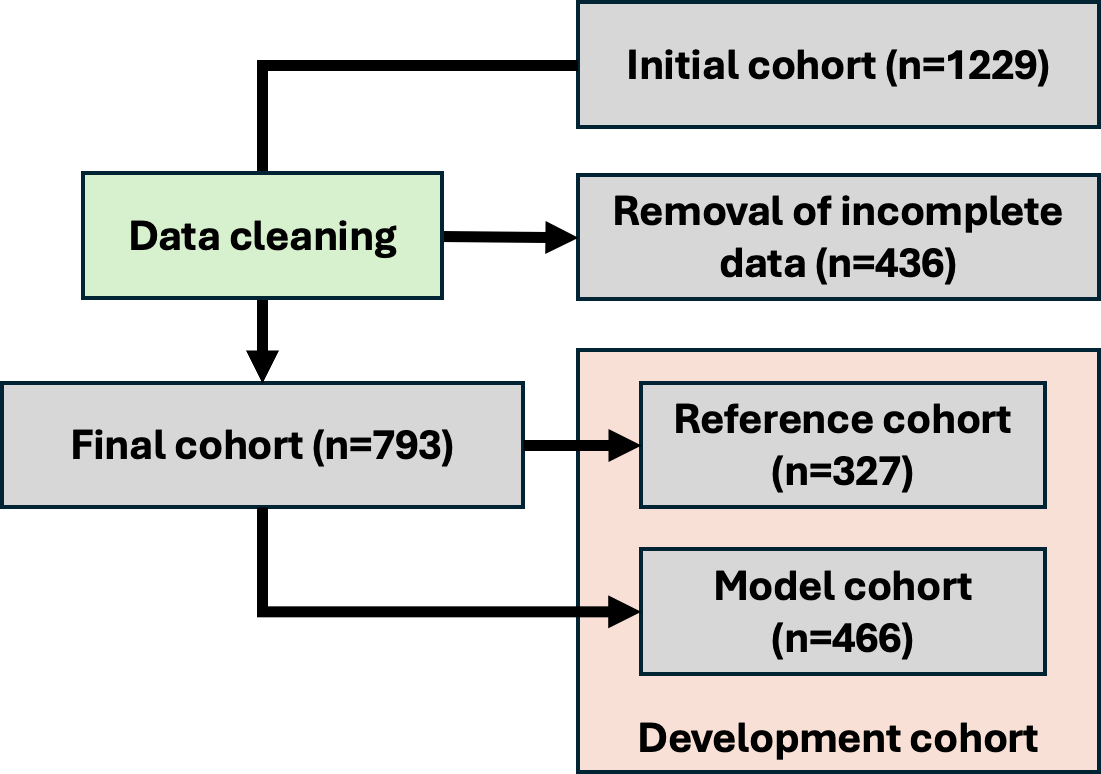
Flowchart of data construction.

A total of 1229 patients comprised the initial cohort. Of these, 793 (65%) remained after entries with missing values were removed (Figure 2). Eliminating the risk of introducing a bias, the initial cohorts’ demographic and clinical characteristics were found to be strictly similar to that of the final cohort and are summarized in Tables1  2.

**Table 1.** Patient characteristics of the initial cohort (N=1229).

Characteristics	Participants
Age at diagnosis (years), n (%)	
70‐74	580 (47)
75‐79	331 (27)
80‐84	204 (17)
85‐89	93 (8)
>90	20 (2)
Performance status, n (%)	
0	339 (28)
1	322 (26)
2	48 (4)
3-4	23 (2)
Missing data	497 (40)
BMI, n (%)	
<18.5	32 (3)
18.5‐25	446 (36)
25‐30	409 (33)
>30	266 (22)
Missing data	76 (6)
Comorbidities, n (%)	
Creatinine clearance <40 mL/minute	57 (5)
Heart failure	105 (9)
Coronary artery disease	123 (10)
Chronic obstructive pulmonary disease	36 (3)
Diabetes	174 (14)

**Table 2.** Cancer characteristics of the initial cohort (N=1229).

Tumor size					
Status	T1	T2	T3	T4	Missing data
Participants, n (%)	567 (46)	286 (23)	36 (3)	250 (20)	90 (7)
Lymph nodes					
Status	N0	N1	N2	N3	Missing data
Participants, n (%)	614 (50)	243 (20)	55 (4)	55 (4)	262 (21)
Grade SBR^a^					
Status	I	II		III	Missing data
Participants, n (%)	188 (15)	648 (53)		281 (23)	112 (9)
Estrogen receptor					
Status	Positive		Negative		Missing data
Participants, n (%)	978 (80)		145 (12)		106 (9)
Progesterone receptor					
Status	Positive		Negative		Missing data
Participants, n (%)	838 (68)		285 (23)		106 (9)

a SBR: Scarff-Bloom-Richardson.

Patient demographics and characteristics were evaluated on the date of breast cancer diagnosis (Table 1). Median age was 75 years (range: 70‐100 years), with 317/1229 (26%) patients aged 80 years or older. Performance status was generally good, as most are categorized as 0 or 1. The main comorbidities were diabetes (174/1229 patients, or 14%), followed by coronary artery disease (123/1229 patents, 10%) and cardiac insufficiency (105/1229 patients, 9%).

The majority presented early-stage tumors (T1 in 567/1229 patients, with a prevalence of 46%), and lymph node involvement was mostly absent (N0 in 614/1229 patients, or 50%). The tumors were typically SBR grade II and 80% (978/1229 patients) were ER-positive. Progesterone receptor positivity was also high at 68% (838/1229 patients). Twelve percent of patients (149/1229) were reported to have received chemotherapy (Table 2).

### Development Cohort

The final cohort was divided into “reference” and “model” cohorts for model development (Figure 2). A total of 327 patients, that is, 50% of patients meeting the criteria for manifold-estimated derivation training were randomly selected. The purpose of this training group was to calibrate the manifold-estimated derivation–scoring algorithm. The model data comprised all remaining patients (466, 59% of the model development cohort).

### Features Performance and Area Under the Curve Scores

**Figure 3.**
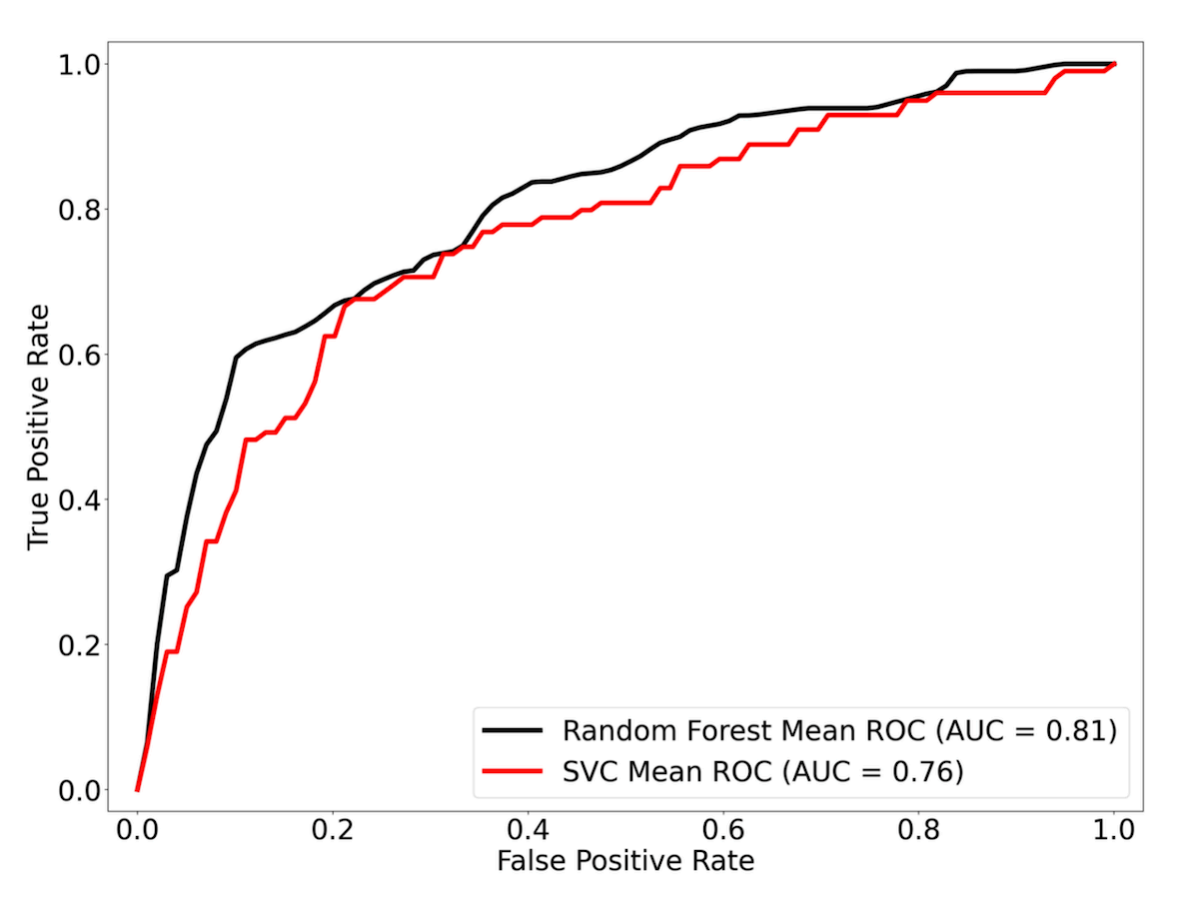
Receiver operating characteristic curves for 5-year mortality predictive models. The predictive efficacy of the selected features was ascertained using Random Forest Classification and Support Vector Classifier. Results are presented as the mean of ROC and AUC values derived from 5-fold cross-validation. AUC: area under the curve; ROC: receiver operating characteristic; SVC: Support Vector Classifier.

In Figure 3, we ascertained the predictive efficacy of the selected variables using RFC and SVC. Analyzing the receiver operating characteristic curves, both models demonstrated commendable predictive capabilities. RFC yielded a mean area under the curve (AUC) of 0.81 (SD 0.06) and a mean accuracy of 0.82 (SD 0.02), while SVC followed closely with a mean AUC of 0.76 (SD 0.05) and a mean accuracy of 0.78 (SD 0.01). The overlapping SDs of these scores suggest that the differences in their performance are not statistically significant.

The overall relative importance of variables for the prediction of the 5-year outcome was also determined by RFC (Table 3). Age, tumor size, and hemoglobin were the top predictors, closely followed by lymphocyte count and BMI. Curiously, the cancer grade, axillary lymph nodes involvement, and the presence of comorbidities ranked low in overall importance. This indicates that although typically taken as important factors from a clinical perspective, comorbidities and cancer grade alone are not the best prognostic features in a patient; rather, a patient’s overall biological profile may be more valuable, underscoring the usefulness of manifold learning as a prognostic tool.

**Table 3.** Overall importance of predictors according to Random Forest Classification.

Variable	Importance (%)
Age	18.33
Tumor size	17.26
Hemoglobin (g/dL)	16.41
Lymphocytes (g/L)	14.84
BMI	13.06
Lymph nodes involvement	10.39
SBR^a^ grade	4.06
ER^b^ status	2.88
Comorbidities	2.78

a SBR: Scarff-Bloom-Richardson.

b ER: estrogen receptor.

### Model Stability

Patients in the test and model groups from the same cluster of origin were compared to evaluate whether data points would exhibit similar distributions (appear in proximity to each other) in the new manifold space across 10 different manifold initializations. The distributions of the test group (n=140) consistently matched closely with those of the model group (n=326), with all *P* values being above the threshold of .05 indicating a lack of significant variation between groups (Figure S1 in Multimedia Appendix 1).

### Prognostic Ability

The primary objective of our study is to evaluate the prognostic ability of the manifold learning model, as measured by the 5-year survival rate of our population. The 3D clusters in Figure 1 illuminated the landscape of our dataset, representing local groups of patients characterized by distinct clinical and prognostic profiles. Clusters are colored based on the overall mortality rate of included patients: Groups 0, 1, and 4 in green have the best prognosis with a 5-year survival rate of more than 80% while group 3 has the worst prognosis with a 5-year mortality rate of at least 35%.

Table 4 further elucidates the variability in values across the patient clusters, especially in BMI, tumor size (in mm), and median age, underscoring the diversity in our cohort.

**Table 4.** Characteristics of the 6 clusters defined by manifold learning.

Feature	Cluster					
	0	1	2	3	4	5
Hemoglobin (g/dL)	13.4	13.3	13	13	13.3	11.9
BMI	25	28.4	24.9	28.9	25.6	23
Lymph nodes involved	0.6	0.8	1.6	6.2	1	0.8
Tumor size (mm)	19.6	19.3	26.1	65.8	23.1	30.6
Age (years)	75.8	76.3	77.8	77.4	79.2	80.5
Lymphocytes (g/L)	1.8	2.1	2.1	1.6	3.4	1.6
Comorbidities	0	1	0.4	0.3	0	0.9
Estrogen receptor status	1	1	0	0.8	1	1
SBR^a^ (high/low)	0	0.1	0.7	0.5	0.8	0.6

a SBR: Scarff-Bloom-Richardson.

### Predictive Ability

**Figure 4.**
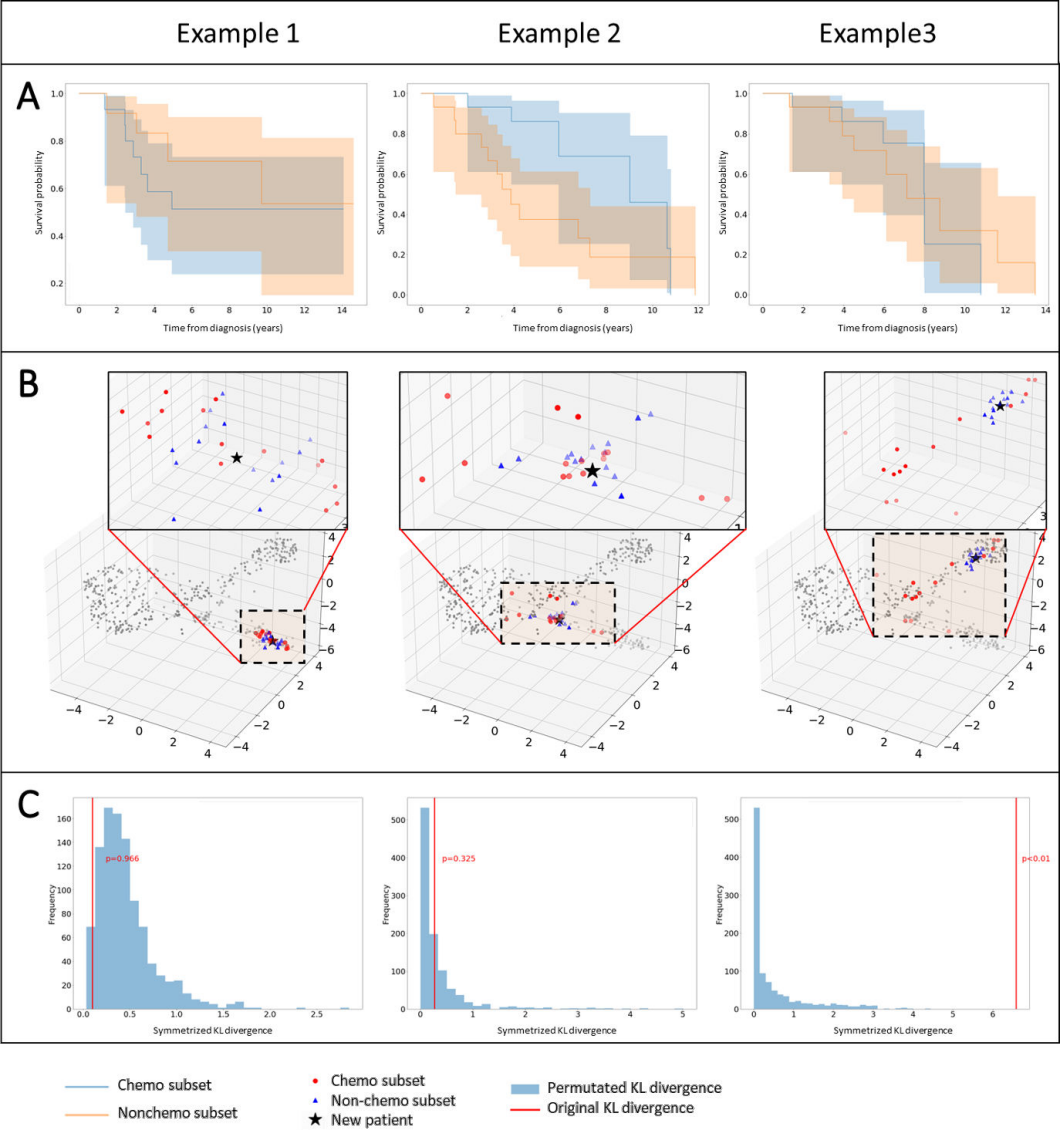
Three case examples assessing the individual benefit of adjuvant chemotherapy. (A) The closest chemotherapy-treated and non–chemotherapy-treated patients to a new patient are identified in the 3D manifold and their survival curves are compared to show the treatment’s potential benefit or lack thereof. (B) The new patient’s position in the 3D manifold (black star), with the 15 closest patients of each treatment groups are shown, displaying varying distributions of treatment subgroups. (C) To quantify distances between the subgroups, the real calculated KL divergence between the treatment groups’ distributions (red line) was compared with that of permutated data (blue histograms) to verify whether observed divergences between treatment subgroups are significant or not. KL: Kullback-Leibler.

Next, we attempted to ascertain the individual benefit of performing adjuvant chemotherapy, demonstrated in Figure 4 with 3 examples.

When a target patient is localized in the 3D manifold, the closest patient profiles are identified. This is done for 2 treatment groups based on whether the patients received chemotherapy (chemo and nonchemo groups), permitting the visualization of KM survival curves that would show the treatment’s potential benefit or lack thereof (Figure 4A).

Figure 4B shows the target patient’s position in the 3D manifold (black star), with the 15 closest patients of each treatment groups also marked. In examples 1 and 2, the 2 treatment groups are found to be well “mixed” in the local vicinity of the target, indicating that the target profile is well represented by similar chemotherapy-treated and non–chemotherapy-treated patients. To quantify distances between the subgroups, we used permutation analysis (Figure 4C). The real calculated KL divergences between the treatment groups’ distributions (red line) for examples 1 and 2 fall well within the range of what could be expected by chance (blue histograms) (*P*>.1), indicating that the observed divergences are not significant.

Example 3 showcases a situation where patients from the 2 treatment groups are not well mixed in the local vicinity of the target patient. In this case, the real KL divergence is far right of the histogram (*P*<.01), suggesting a significant difference between the distributions. Thus, the KM survival curves and any conclusion drawn from them must be taken with consideration of the heterogeneity in the profiles of the treatment groups being compared.

## Discussion

### Principal Findings

From an initial cohort of 1229 patients, we used 793 (65%) to develop a model that clustered patients by their clinical and biological features. These clusters represent a potential prognostic tool for physicians, attributing a risk of mortality in 5 years to patients with consideration to multivariate profiles. The model is further able to indicate the potential benefit or lack thereof of chemotherapy treatment in older adult patients. We found that the predictors used in our model gave a good overall result of 0.81 and 0.76 AUCs with RFC and SVC, respectively.

In summation, our multifaceted approach, blending manifold learning with classical machine learning paradigms and intuitive data visualizations, has unveiled profound insights into the prognosis determinants of early-stage breast cancer in older adults. These revelations bring a more nuanced understanding of the disease and hold promise for tailoring patient-specific therapeutic strategies. Our study’s utilization of manifold learning and advanced machine learning algorithms represents a significant contribution to oncology. The accuracy of 81% in differentiating patient subgroups through manifold learning is impressive, showcasing an advancement beyond traditional linear models [33]. This approach is in line with recent trends in personalized medicine [34,35], which discuss the potential of machine learning in cancer prognosis. The high AUC values achieved by RFC and SVC reflect the importance of our combined predictors in medical diagnostics, aligning with the findings of recent studies on the application of machine learning in cancer detection [36,37]. The application of data visualization techniques such as heatmaps and 3D scatterplots in elucidating complex clinical relationships is noteworthy. This approach is supported by advancements in data visualization in medical research, as seen in the study by Borkin et al [38] on how data visualization supports medical decision-making [39,40].

### Limitations

The present results should be interpreted in the light of some limitations. First, the monocentric nature of the research may impact the representativeness of the cohort, potentially affecting the generalizability of our findings. Second, the exclusion of specific patient characteristics, such as the ONCODAGE score [41], from our datasets may have limited the comprehensiveness of our prognostic tools. Third, the retrospective design of the study constrains our ability to establish causality between clinical characteristics and patient outcomes. A fourth limitation concerns the fact that patients may present with or have a history of multiple comorbidities. We chose to group together patients with any number of comorbidities for reasons related to (1) the reduction of the sample size for each category of comorbidity, and (2) the potential skewing of patient distribution in the 3D manifold due to multiple related qualitative variables. PaCMAP is susceptible to “overseparate” the population if provided with too many binary features. These reasons in mind, we nonetheless acknowledge that omitting the consideration of multiple comorbidities is a limitation of the study. Other notable limitations include the absence of cancer-specific or treatment-specific survival metrics, a lack of detailed analysis on specific comorbidities, and the need for more data to enhance the less populated clusters. Furthermore, the external validation of our model remains pending, which is crucial for assessing its generalizability.

### Future Prospects

Looking forward, the promising application of manifold learning in oncology, as demonstrated in our study, aligns with the burgeoning field of personalized medicine. The integration of machine learning in personalized cancer therapy, as discussed by Danishuddin et al [42], supports the potential of such approaches. The development of advanced AI-driven prognostic tools, particularly for older adult patients who are often underrepresented in clinical trials, could revolutionize treatment guidelines and care approaches. The rapid advancement of machine learning techniques poses a challenge in ensuring the longevity and relevance of models, necessitating continuous updates. This is echoed in the broader context of AI in health care, as discussed in Topol’s [43] comprehensive review of AI in medicine. Concerns about the adoption of AI tools due to accuracy, explainability, and ethical considerations are also prevalent, as reflected in the exploration of implementing AI in clinical practice by Char et al [44]. Our findings may open up avenues for the personalized treatment specifically catered to neglected populations in oncology, starting with geriatric patients with breast cancer. We expect our software to provide rapid guidance to physicians in the process of charting treatment plans for their patients, going beyond simple monovariate statistics and instead considering patients’ combined clinical and biological profiles.

### Conclusions

Our study aimed to further the management of early breast cancer in older adult patients by integrating cutting-edge AI techniques. We proposed a technique that uses patient data to create a visualizable 3D map of pathology profiles that allow rapid prognostic estimations for new patients. These prognostic predictions include the potential benefits of treatment strategies such as chemotherapy, aiding clinical decision-making. It reflects the ongoing evolution in oncology, emphasizing the importance of tailored treatment strategies and highlighting both the potential and the challenges of AI applications in health care. This study also prompts considerations for future research directions and ethical implications in the rapidly evolving field of AI in medicine.

## Supplementary material

10.2196/64000 Multimedia Appendix 1 Stability analysis of manifold learning applied to clustered data. The original cohort data were divided into 2 groups; 70% of every cluster was pooled into the model group, and the remaining 30% was pooled into the test group. A fresh manifold learning process was applied to the model group, and the test group was then projected onto the newly generated manifold. Patients in the test and model groups from the same cluster of origin were compared to evaluate whether they would exhibit similar distributions (appear in proximity to each other) in the new manifold space. (A) Examples of permutation analysis of clusters 0 and 1. The permutation test determined whether the observed KL (red line) divergence was significantly different from what can be expected from random shuffling of the 2 groups (blue histograms). (B) Table summarizing the median *P* values of the stability tests across 10 different manifold initializations. All *P* values above .05 indicated a lack of significant variation between groups.

## Abbreviations

AI: artificial intelligence
AUC: area under the curve
COPD: chronic obstructive pulmonary disease
ER: estrogen receptor
HER2: human epidermal growth factor receptor 2
KL: Kullback-Leibler
KM: Kaplan-Meier
PaCMAP: Pairwise Controlled Manifold Approximation
PR: progesterone receptor
RFC: Random Forest Classification
SBR: Scarff-Bloom-Richardson
SVC: Support Vector Classification
