# Supplementary figures and images for: Leveraging Digital Twins for Stratification of Patients with Breast Cancer and Treatment Optimization in Geriatric Oncology: Multivariate Clustering Analysis
Source: JMIR Cancer. 2025 May 23;11:e64000. doi: 10.2196/64000 (PMC12124816; doi:10.2196/64000)

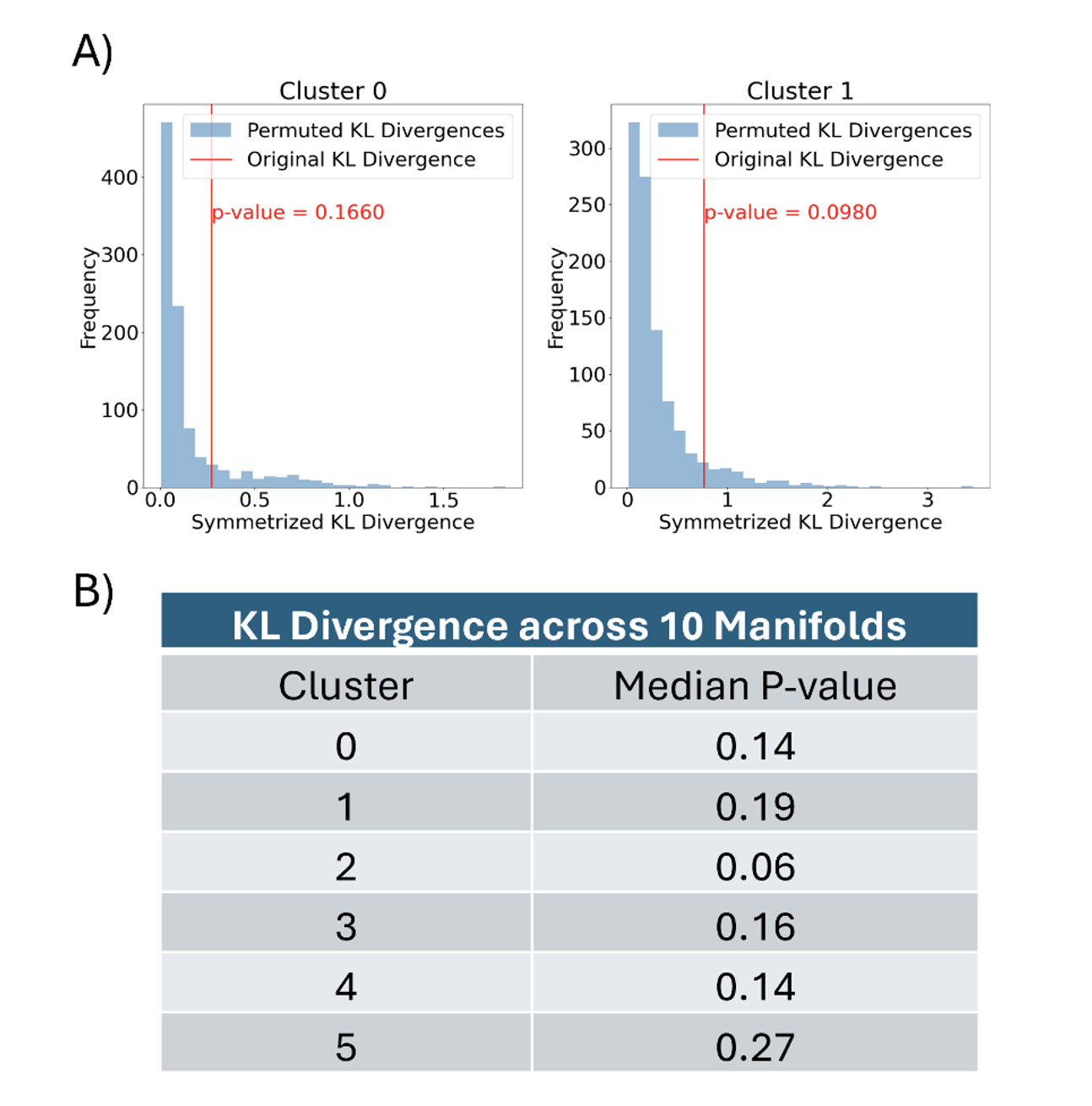

Supplement: Multimedia Appendix 1 [file cancer-v11-e64000-s001.png]
